# PTB Binds to the 3’ Untranslated Region of the Human Astrovirus Type 8: A Possible Role in Viral Replication

**DOI:** 10.1371/journal.pone.0113113

**Published:** 2014-11-18

**Authors:** Wendy Espinosa-Hernández, Dora Velez-Uriza, Jesús Valdés, Cristina Vélez-Del Valle, Juan Salas-Benito, Rebeca Martínez-Contreras, Matilde García-Espítia, Mariana Salas-Benito, Tania Vega-Almeida, Mónica De Nova-Ocampo

**Affiliations:** 1 Programa Institucional de Biomedicina Molecular, Sección de Estudios de Posgrado e Investigación, ENMH, Instituto Politécnico Nacional, Col. Fracc. La Escalera-Ticomán, México D.F., México; 2 Departamento de Bioquímica, Centro de Investigación y de Estudios Avanzados del IPN, Col. San Pedro Zacatenco, México D.F., México; 3 Departamento de Biología Celular, Centro de Investigación y de Estudios Avanzados del IPN, Col. San Pedro Zacatenco, México D.F., México; 4 Centro de Investigaciones en Ciencias Microbiológicas, Edificio 103, Instituto de Ciencias, Benemérita Universidad Autónoma de Puebla (BUAP), Col. San Manuel, Puebla, México; 5 Facultad de Medicina, Departamento de Microbiología y Parasitología, Universidad Nacional Autónoma de México, Circuito interior, Ciudad Universitaria, México D.F., México; International Centre for Genetic Engineering and Biotechnology, Italy

## Abstract

The 3′ untranslated region (3′UTR) of human astroviruses (HAstV) consists of two hairpin structures (helix I and II) joined by a linker harboring a conserved PTB/hnRNP1 binding site. The identification and characterization of cellular proteins that interact with the 3′UTR of HAstV-8 virus will help to uncover cellular requirements for viral functions. To this end, mobility shift assays and UV cross-linking were performed with uninfected and HAstV-8-infected cell extracts and HAstV-8 3′UTR probes. Two RNA-protein complexes (CI and CII) were recruited into the 3′UTR. Complex CII formation was compromised with cold homologous RNA, and seven proteins of 35, 40, 45, 50, 52, 57/60 and 75 kDa were cross-linked to the 3′UTR. Supermobility shift assays indicated that PTB/hnRNP1 is part of this complex, and 3′UTR-crosslinked PTB/hnRNP1 was immunoprecipitated from HAstV-8 infected cell-membrane extracts. Also, immunofluorescence analyses revealed that PTB/hnRNP1 is distributed in the nucleus and cytoplasm of uninfected cells, but it is mainly localized perinuclearly in the cytoplasm of HAstV-8 infected cells. Furthermore, the minimal 3′UTR sequences recognized by recombinant PTB are those conforming helix I, and an intact PTB/hnRNP1-binding site. Finally, small interfering RNA-mediated PTB/hnRNP1 silencing reduced synthesis viral genome and virus yield in CaCo2 cells, suggesting that PTB/hnRNP1 is required for HAstV replication. In conclusion, PTB/hnRNP1 binds to the 3′UTR HAstV-8 and is required or participates in viral replication.

## Introduction

Human astroviruses (HAstVs) are a major cause of acute gastroenteritis in children constituting an important public health problem. The astroviruses belong to the *Astroviridae* family, consisting of non-enveloped viruses whose plus-sense, single-stranded polyadenylated genome of approximately 7 kb, is organized in three open reading frames (ORFs) ORF1a, ORF1b and ORF2 [1], [2], preceded and followed, respectively, by the 5′ and 3′ untranslated regions (UTRs) of 80 to 100 nucleotides (nt) [3]. It has been suggested that RNA viral replication occurs in the endoplasmic reticulum-derived intracellular membrane, and is mediated through interactions between the ORF1A-encoded nsP1a/4 phosphoprotein - which includes residues of the viral genome-linked protein (VPg) - and the RNA-dependent RNA polymerase (RdRp)-encoding ORF1b [4]–[7].

Viral 3′UTRs participate in the interaction with host-cell and viral proteins, and may interact with other parts of the viral genome [8], thus contributing to host specificity, tropism and pathogenesis. In spite of this, only one report exists regarding the RNA secondary structure of the 3′ end of HAstV-1, which is similar to those of other RNA viruses [9], and little is known about the specific interactions involved in viral 3′UTR recognition and the underlying molecular mechanisms. The RNA elements within the highly conserved 3′UTR may help recruit the cellular factors that mediate viral translation and replication [10].

In other single-stranded positive RNA viruses, the interaction between the UTRs and cellular factors such as Polypyrimidine Tract Binding protein (PTB, also referred as hnRNP1), several heterogeneous nuclear ribonucleoproteins (hnRNPs), La autoantigen (La), poly (A) binding protein (PABP), poly-r (C)-binding proteins (PCBP1/2) also known as hnRNPE2, cellular protein eukaryotic elongation factor 1A (eEF1A) and Nucleolin, might be involved in viral replication [10]–[17]. In addition, some viral proteins are also recruited to the UTRs in order to form a functional viral replication complex (VRC) on subcellular membrane surfaces [18], [19]. The role of these proteins in viral replication has been depicted, but the precise mechanism underlying the function of all viral and cellular proteins involved has not been fully unraveled. For example, some might be involved in membrane remodeling, creating a favorable microenvironment for viral replication, others as chaperones that facilitate proper folding during VRC assembly and function [19].

The 3′UTR of all HAstV serotypes is highly conserved. As predicted in silico, the RNA structures these 3′UTRs are conformed by two hairpins joined by a single stranded linker, and a putative PTB binding site located either within or next to the linker. This structural conservation suggests that the 3′UTR might be involved in the regulation of viral activities, such as replication. To explore the role of the HAstV-8 3′UTR RNA in the regulation of viral functions, we studied the interaction of human CaCo2 cellular factors with RNA probes of this region with or without the PTB binding site. Mobility shift assays evidenced the formation of two RNA-protein complexes, CI and CII, and the supershift of complex CII by PTB antibodies. UV cross-linking assays showed that seven proteins of 35, 40/45, 50, 52, 57/60 and 75 kDa were directly bound to this 3′UTR, and immunoprecipitation assays identified PTB in the 57/60-kDa bands. Also, immunofluorescence analysis showed perinuclear PTB signals localized mainly in the cytoplasm of HAstV-8 infected cells. Moreover, recombinant PTB was cross-linked to 3′UTR probes containing intact helix I, and PTB binding sites. As expected, siRNA-mediated PTB knockdown in HAstV-8 infected CaCo2 cells showed reduced RNA viral genome synthesis and viral yield. Our results suggest that PTB binds to the cognate UCUU site, localized in the linker of the 3′UTR of HAstV-8, that helix I is also required for such binding, and that it is required and participates in viral replication.

## Materials and Methods

### Cells and virus

Monolayers of CaCo2 cells (human adenocarcinoma HTB-37, ATCC) were grown at 37°C, 5% CO_2_ in Advanced-Dulbecco’s modified Eagle’s medium (DMEM) (Invitrogen) supplemented with 5% fetal calf serum (FCS) (Biowest, France Labs), antibiotics (penicillin and streptomycin) and 2 mM L-Glutamine (Invitrogen). CaCo2 cells were infected with HAstV-8 strain Yuc 8 (kindly provided by E. Mendez, IBT, UNAM, México). The Infections and the virus were obtained as previously described [20].

### Preparation of cell extracts

Cytoplasmic S10 fractions or 1% NP-40 cytoplasmic membrane extracts were obtained from uninfected and HAstV-8-infected CaCo2 cells as previously described [21], [16]. The extracts were aliquoted, and proteins were quantitated with the Bradford assay kit (Sigma) following manufacturer’s instructions. Extracts were stored at −80°C until use.

### Western-blotting of cell extracts

Thirty or sixty micrograms of cell extracts were monitored by western-blotting. To rule out cross-contamination between cytoplasmic, membrane and nuclear fractions, a monoclonal anti-Actin (Millipore), polyclonal anti-Lamin A and polyclonal anti-hnRNP1 (Santa Cruz Biotechcnology) were used. PTB was monitored with a polyclonal anti-hnRNP1 and monoclonal anti-carboxy-terminus PTB (Invitrogen). The cytoplasmic membrane-associated proteins were monitored with polyclonal anti-PDI (Cell Signall). Proteins were developed by chemiluniscense following the manufacturer’s instructions (SuperSignal, West Pico, Pierce).

### PCR amplifications

Oligonucleotides (Invitrogen; all 5′→3′) were used amplify by PCR the full-length 3′UTR and mPTB templates, and their short versions HI and HI/mPTB. DNA templates for the full length-3′UTR, mPTB and HI/mPTB were generated by DNA duplexes according to the protocol recommended by Sigma-Aldrich, using the respective primer sets HAstV-8-F/HAstV-8-R (spanning nt 6674 to 6759 from strains Yuc8; accession AF260508), mPTB-fwd/mPTB-rev (UCUU PTB binding site within nt 6700 to 6704 changed to CGAA), and HI/mPTB-fwd (TAATACGACTCACTATAGGG GAAGGAGGGTACAGCTTCCTATCCTCGAATTC)/HI/mPTB-rev (GAATTCGAGGATACGAAGTGTACCCTCCTTC). These products were used as templates to amplify the PCR templates RNA synthesis, including the full-length 3′UTR and mPTB (primers HAstV8s-fwd TAATACGACTCACTATAGGG GATCGAGGG, and HAstV8s-rev GCATCTGATTAAATC), as well as the 32 bp-long HI (6674–6706 nt; primers HAstV8s-fwd and HI-rev: GAAAAGAAGGATAG) oligonucleotides. To maintain the same structure of the wt probe, nucleotides T_6676_C_6677_ were substituted for AG in the HI/mPTB template. The T7 RNA polymerase promoter (underlined) was included in all forward oligonucleotides used. DNA duplexes were visualized on 8% native acrylamide gel before use for PCR amplification. PCR reactions were done in presence of 10 µM of DNA duplex for full-length 3′UTR, HI/mPTB, and mPTB, whereas HI used the full-length 3′UTR as a template. The PCR mix contained 10 µM of each oligonucleotide, 10 mM dNTPs (Roche), 25 mM MgCl_2_, and 2 U of Dream Taq polymerase in the appropriate buffer (Fermentas). Incubation times and temperatures were: 2 min at 94°C, followed by 35 cycles of 1 min at 94°C denaturation, annealing for 1 min at 52°C and extension 1 min at 72°C, and a final extension at 72°C for 5 min. The same conditions were used for the unrelated RNA (REST) control template with the primers reported elsewhere [22]. Amplification of the expected products was monitored on an ethidium bromide-2% agarose gel.

### Synthesis of RNA probes

DNA templates were used to synthesize UTP-^32^P (NEN-Dupont)-labeled RNA transcripts with RiboProbe T7 Transcription System (Promega) following the conditions described by the manufacturer. One unit of DNase I- RNase-Free (Promega) was added for 30 min at 37°C and transcripts were purified with NucAway columns (Ambion) as recommended by the manufacturer. The RNA transcripts were measured in a Beckmann scintillation counter and stored at −20°C until used. The unlabeled probes were synthesized with MegaShort-Script T7 Transcription kit (Ambion), as recommended by the manufacturer.

### Mobility shift assays

Five and ten micrograms of S10 extracts from uninfected and HAstV-8 infected CaCo2 cells were use for mobility shift assays following protocols previously described [15] with modifications. Labeled RNA (5×10^4^ cpm) was added and the mixture was incubated at 4°C for 15 min to allow complex formation. Gels were dried and autoradiographed. For the competition assays, unlabeled RNAs were included in the preincubation reaction. For super mobility shift assays, polyclonal antibodies to hnRNP1 and goat-IgG anti-rabbit-HRP (Zymed) non-related antibodies were preincubated for 15 min in the binding reaction after the addition of labeled RNA.

### UV crosslinking and immunoprecipitation assays

The UV crosslink assays were carried out using protocols previously described [15] with modification. Briefly, each ^32^P-labeled RNA probe (1×10^5^ cpm) was incubated at 4°C in crosslink binding buffer (50 mM HEPES pH 7.9, 80 mM MgCl_2_, 0.5 mM DTT, 124 mM KCl, 20% Glycerol) with S10 extracts (20 µg) from the uninfected and HAstV-8 infected CaCo2 cells or purified recombinant PTB (0.50 and 1 µg). RNA-protein complexes were irradiated with UV light (254 nm) for 20 min on ice and the unbound RNA was digested with RNase A and RNase T1 (20 µg and 20 U, respectively) for 30 min at 37°C. Samples were analyzed by 10% SDS-PAGE. Gels were dried and autoradiographed. UV crosslinking- immunoprecipitations were carried out as described previously [23], with some modifications. 2 µg of anti- hnRNP 1 or 1 µg of non-related antibodies were immobilized on protein-G Sepharose (Sigma) for 2 h at 4°C followed by centrifugation at 12,000 rpm for 15 sec. The unbound antibodies were removed by three washes with IPP buffer (10 mM Tris-HCl pH 8, 150 mM NaCl, 0.1% NP40). The UV crosslink samples were incubated overnight at 4°C with a corresponding immobilized protein-G-antibody. Unbound material was removed as above. Bound proteins were analyzed as described above.

### siRNA-mediated knockdown of PTB, qRT-PCR and viral titration

For siRNA-mediated knockdown of PTB expression, CaCo2 cells were transfected using the Nucleofector device and solution T, as recommended by the manufacturer (Amaxa VCA-1002, Lonza). For each siRNA, the optimal concentrations were determined experimentally and all were obtained from Ambion. Mock-transfected cells were treated with transfection reagent only, and a nontarget siScrambled (160 nM) was used as a negative control. The concentrations used for siPTB were 2.5 µM and 5 µM. 72 h post- transfection, the cells were infected with HAstV8, and samples were harvested for RNA isolation, western-blotting and virus titer determination at 15 h p.i. For RNA and Proteins were isolated with AllPrepRNA/Protein kit (Qiagen) as recommended by the manufacturer. Proteins were quantitated with 660 nm Protein Assay Reagent (Pierce) following manufacturer’s instructions and cellular and viral protein expressions were determined by western-blotting with anti-hnRNP1, viral RdRp anti-1b-2 (kindly provided by E. Mendez, IBT, UNAM, México) as previously described [20] and anti-Actin polyclonal antibodies (Santa Cruz, Biotechnology). Densitometry was performed with Gel Quant Express 3.1 software (2005) DNR Ltd and ImageJ1.48a software (National Institute of Health, USA). The relative RNA level of HAstV8 was evaluated by quantitative RT-PCR. The conserved ORF1a region was analyzed (nt 1182–1470) and simultaneously expression of the glyceraldehydes-3-phosphate dehydrogenase (GAPDH) gene as a control. Two hundred nanograms of RNA was reverse transcribed using Transcriptor First Strand cDNA Synthesis kit (Roche) according to the manufacturer’s instructions with 25 pmol of Mon348 oligonucleotide in order to mainly detect antigenomic RNA [24], [52]. The cDNA was quantified using the GloMax Multi Detection System device. 500 ng of cDNA was used by the PCR step in a final reaction volume of 20 µl with 2X KAPA SYBR FAST qPCR Master Mix2 Universal, 25 pmols each Mon 340 and Mon 348 oligonucleotides and for GAPDH-F (CATCTCTGCCCCCTCTGCTGA) and GAPDH-R (GGATGACCTTGCCCACAGCCT). Incubation times and temperatures were: 10 min at 95°C, followed by 30 cycles at 95°C for 30 sec, 50°C for 30 sec and 72°C for 30 sec. The reaction was performed three times for each sample using a Rotor Gene 6000 (Corbett Research). The data were analyzed with the comparative threshold cycle (2-ΔΔC_T_) method. The virus titer was reported as focus-forming units/ml [20], [25], [26]. The two- tailed Student *t* test was used to compare between two treatment groups. The *P*-values<0.005 were considered as statically significant. All statical analyses were done using GraphPad Prism 6 (GraphPad Software Inc, La Jolla, CA).

### Immunofluorescence

Immunofluorescence assays were carried out as previously described [26] with modifications. CaCo2 cells (0.4×10^5^) were seeded in an 8-well chamber slide plate (Chamber-Slide System, Nunc) and grown at 37°C, 5% CO_2_, 72 h. The anti-CQFG antibody, which recognizes the VP34 capsid protein (a generous gift of E. Mendez, IBT, UNAM, México), was used (1∶100 dilution) to monitor HAstV8 infection of CaCo2 cells. PTB silencing was monitored with the anti-hnRNP1 antibody. For immunofluorescence, cells were washed in PBS- 50 mM NH_4_Cl, incubated with secondary antibodies anti-rabbit coupled to Alexa-Fluor 594 at 1∶700 dilution (Invitrogen), Fluorescein (FITC)-conjugated donkey anti-goat IgG at 1∶75 dilution (Jackson ImmunoResearch), respectively 1 h at room temperature. Finally, the cells were incubated with Vectashield with DAPI mounting media (Vector Laboratories, Inc.), and visualized under lasser confocal microscopes LSM710 (Carl Zeiss) and Olympus Fv300.

### Secondary structure predictions of HAstV 3′UTRs

The secondary structure modeling of the full-length 3′UTR sequences of HAstV-1 to −8 (Accession Nos. Z25771, AF141381, AY720891, DQ028633, Y08632, and AF260508) were carried out with the RNA Mfold software release (version 3.2) [27]. The targets for the putative binding sites for cell factors were predicted utilizing the ESEfinder 3.0 [28], [29], the ESRsearch [30], [31], and the EBI-EMBL ASD-Alternative splicing search engines.

## Results and Discussion

### Several cellular proteins bind to the 3′UTR of HAstV-8 RNA

**Figure 1:**
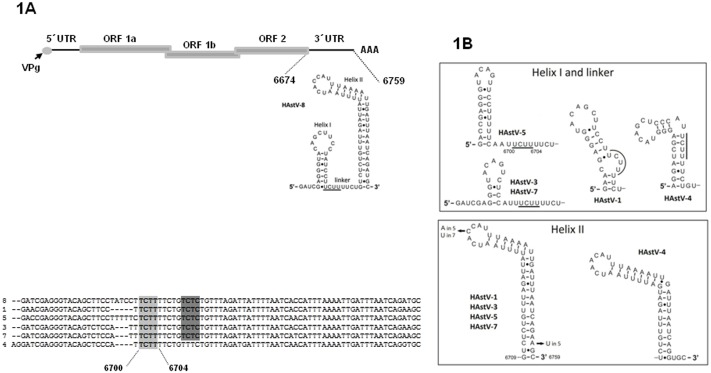
CaCo2 cellular proteins interact with the HAstV-8 3′UTR. **A)** Diagram of the HAstV-8 genome. The blowout represents the 3′UTR, showing the minimal energy model of its secondary structure. The 3′UTRs of all astrovirus contain one consensus UCUU PTB binding site (light gray) and a second UCUC element PTB (dark gray). Hyphens indicate gaps in the sequence. **B)** The RNA structures of the viral 3′UTR of all HAstVs were predicted with the MFold software. Comparisons of Helix I-linker structures and of Helix II structures are shown in the upper and lower panels, respectively. Residues within the single stranded regions of the putative PTB binding sites appear underlined.

The integrity of the secondary structure of the 3′UTR facilitates the recruitment of cellular and viral factors to the VRC and is also involved in viral viability, RNA stability, translation initiation, or intracellular localization [32]–[35]. Thus far, only the structure of HAstV-1 3′UTR RNA has been described only [9], [8], therefore we analyzed the predicted structures of as many HAstV sequences available (Fig. 1A). Due to its prevalence in México, we focused on HAstV-8 3′UTR structure. It is arranged in two helixes (I and II) connected through a linker (Fig. 1A); splitting the 3′UTR facilitated comparisons between strains. Between serotypes, linker lengths are the source of variation in the helix I-linker regions, albeit in helix II nucleotide variations were found (Fig. 1B; nucleotide changes are shown for each strain). Putative PTB/hnRNP1 binding sites UCUU are found in the single-stranded (or partly) linkers of HAstV-3, -5, and -7, whereas in HAstV-1 and -4 these elements are localized nearby. At the beginning of helixes II (Fig. 1A), all but HAstV-4 contain the previously reported PTB/hnRNP1 binding element CUCUCU [36]. This structural arrangement could partially explain the prevalence of serotypes in the world [8], probably as a consequence of the interaction with cellular and viral factors that may impact viral functions [37].

**Figure 2:**
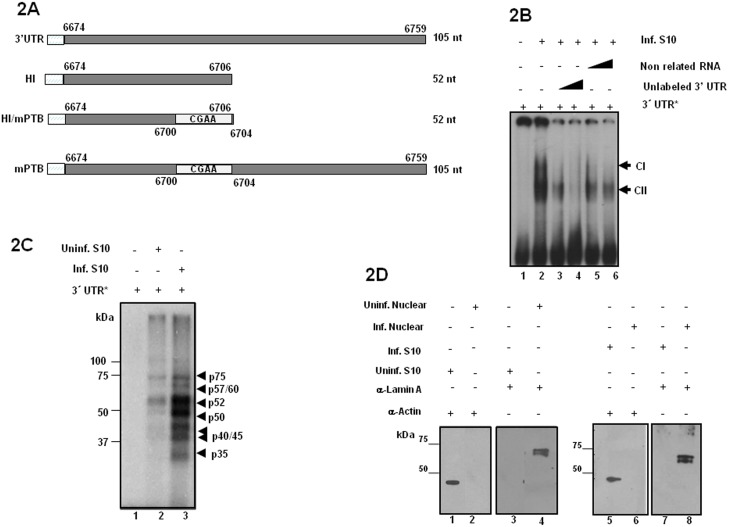
CaCo2 cellular proteins interact with the HAstV-8 3′UTR. **A)** Schematic representation of the 3′UTR from HAstV-8 and their short versions used to produce RNAs. The complete 3′UTR and mPTB version consisting of nt 6,674 to 6,759, H1 and HI/mPTB shorts versions consisting of nt 6,674 to 6,706. The versions HI/mPTB and mPTB has a UCUU PTB binding site within nt 6700 to 6704 changed to CGAA. The amplicons were used for RNA probe synthesis. **B)** Competition gel mobility shift assay. Different amounts of non-radiolabeled competitor RNA were incubated with 5 µg of S10 extracts from HAstV-8-infected CaCo2 cells before addition of the labeled 3′UTR RNA. Free probe (lane 1); (lane 2) no competitor; 20-fold (lanes 3 and 5) or 60-fold molar excess (lanes 4 and 6); (lanes 3 and 4) cold homologous RNA or heterologous RNA (lanes 5 and 6). Complex CI and specific complex CII are indicated. **C)** Labeled 3′UTR RNA (nt 6674 to 6759) was incubated without extract (lane 1) or with 20 µg of S10 from uninfected (lane 2) or HAstV-8-infected CaCo2 cells (lane 3). After UV crosslinking, the proteins were separated by SDS-10%PAGE and detected by autoradiography. Arrows indicate cross-linked proteins with the molecular masses in kilo daltons. **D)** Thirty micrograms of uninfected (U) and infected CaCo2 cells cytoplasmic and nuclear cell extracts were analyzed by western blotting with monoclonal anti- Actin and polyclonal anti-Lamin A antibodies.

Cellular proteins bound to the 3′UTR in positive-strand RNA viruses, play important roles in assembling the viral RNA replication complex, selecting and recruiting viral RNA replication templates to perform the synthesis of the minus-strand RNA [10], [19]; among them are PTB, EF-1α , La, Nucleolin, Sam68, PABP, PCBP1/2 and TIA-1/TIAR [38]–[40], [15], [41]–[43], [19], [17]. Mobility shift assays showed that two protein complexes (CI and CII) were recruited on the HAstV-8 3′UTR RNA, with two different amounts of uninfected and infected CaCo2 cells extracts (data no shown). The specificity of the interactions between the HAstV-8 3′UTR and infected cells extracts was analyzed in a competition assays using 20-fold and 60-fold molar excess of unlabeled homologous RNA 3′UTR as a competitor. The cellular proteins-viral RNA Complex CII was competed with cold homologous RNA (Fig. 2B), indicating that RNA binding of these proteins was specific and probably through PTB/hRNP1 binding sites. In contrast, in complex CI the binding of cellular proteins to the 3′UTR RNA sequences seems to be non-specific, since it was competed both by homologous and heterologous RNAs, without PTB/hnRNP1 binding sites. Therefore, RNA-protein interactions in CI are likely mediated by motifs other than the PTB/hnRNP1 binding site.

When labeled HAstV-8 3′UTR (nt 6674 to 6759) was UV cross-linked to uninfected and infected CaCo2 cell extracts we detected that seven proteins of 35, 40, 45, 50, 52, 57/60 and 75 kDa from both cell extracts were bound to the probe. However, stronger signals of individual proteins of infected cell extracts were observed (Fig. 2C, lanes 2 and 3, respectively), reflecting the presence of infection-specific factors resulting in more efficient binding of proteins to the 3′UTR. Except for the 75 kDa protein, proteins of similar molecular weight were previously reported. To exclude cross-contamination between cytoplasmic and nuclear fractions, western blots were carried out with anti-Actin and anti-Lamin A antibodies (Fig. 2D). As expected, the cytoplasmic fractions were free of nuclear proteins contamination (compare lanes 1, 3, 5 and 7).

### PTB/hnRNP1 is a component of the HAstV-8 3′UTR RNA-protein complexes

**Figure 3:**
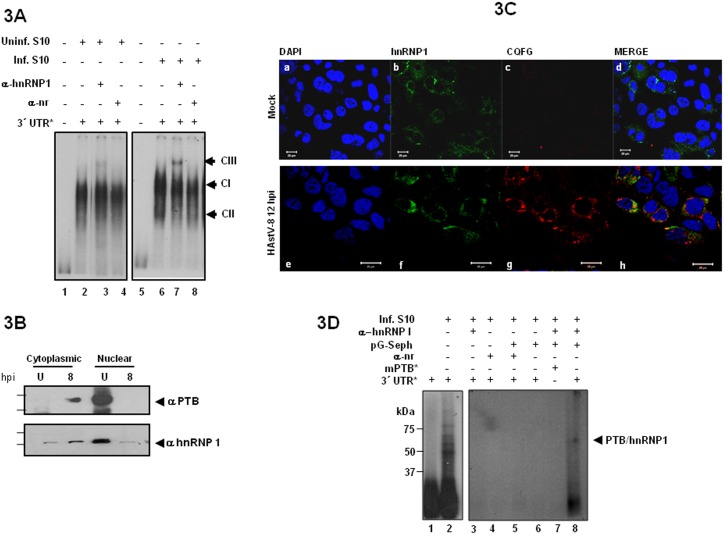
PTB bind to the HAstV8 virus 3′UTR. **A)** Supermobility shift assay from uninfected or HAstV-8 infected CaCo2 S10 extracts were incubated without (lane 2) or with anti-hnRNP1 (lane 3) or with a non-related antibody (lane 4) after of the 3′UTR labeled RNA (lane 1). Supershifted complexes are indicated as CIII. **B)** Uninfected (U) and infected CaCo2 cells cytoplasmic and nuclear cell extracts (8 h p.i.) prepared as described in Figure 2D were probed with monoclonal anti-PTB and polyclonal anti-hnRNP1 antibodies by western blotting. **C)** Colocalization of hnRNP1 (green) in Mock infected (upper panel) or infected CaCo2 cells (lower panel). Cells were fixed at 12 h p.i. and were subjected to indirect immunofluorescence. The HAstV-8 infected positive cells were labeled with anti-HAstV CQFG sera (red). Nuclei are shown by DAPI (blue) staining. The images of cell were acquired with a confocal lasser scanning microscope LSM 710 (Carl Zeiss). **D)** 3′UTR RNA was cross-linked with 60 µg of HAstV-8-infected CaCo2 cells extracts (lanes 2 to 6 and 8) or mutant mPTB RNA labeled (lane 7) and immunoprecipitated with anti-hnRNP1 antibody (lanes 7 and 8) or with a non-related antibody (lane 5). The lanes 4 and 6 are internal controls. Probes are indicated with asterisks and the arrow indicates the position of the immunoprecipitated labeled PTB/hnRNP1.

Supershift assays were conducted to confirm the identity of the 57/60 kDa UV cross-linked protein (Fig. 2C). Incubation of anti-hnRNP1 antibody with cytoplasmic uninfected and infected cell extracts (Fig. 3A) after the addition of the labeled 3′UTR RNA, resulted in the formation of an additional supershifted CIII complex. Complex CIII was absent in the control reactions without antibody and with an unrelated antibody (lanes 2, 4, 6 and 8 in Figs. 3A). Apparently complex CIII is slightly enriched in infected cells suggesting that PTB/hnRNP1 is present in the complexes recruited in the 3′UTR RNA. To exert its role in viral processes, upon viral infections, nuclear PTB/hnRNP1 shuttles rapidly to the cytoplasm, retaining its predominant nuclear localization [44]. For example, during dengue and feline calicivirus infections, PTB/hnRNP1 shuttles to the cytoplasm and participates in viral replicative cycles of both viruses [45], [46]. PTB/hnRNP1 is maintained in the cytoplasm upon cellular stress, such as viral infections [44], [50]. In steady state cells, PTB/hnRNP1 also functions in mRNA transport and stability as well as IRES-mediated translation. Cytoplasmic localization is achieved mainly by PKA-mediated phosphorylation of Ser-16 within the nuclear localization signal. To further confirm the sub-cellular distributions of PTB/hnRNP1 during HAstV-8 infection, western blot and co-localization assays were carried out with anti-hnRNP1 and anti-PTB antibodies (Fig. 3B and C). Monoclonal antibodies against the RNA binding domain (C-terminus) of PTB showed that in HAstV8 infected cells, nuclear PTB/hnRNP1 moves out to the cytoplasm (Fig. 3B). Similarly, the polyclonal anti-hnRNP1 antibody, against the nuclear localization and export signals – NLS and NES – (N-terminus) of PTB/hnRNP1, also shows the same nuclear-cytoplasm shuttling of PTB/hnRNP1. Due to the difference of epitope recognition this antibody was also useful in detecting the nuclear and cytoplasmic localizations of PTB/hnRNP1, regardless of infection. Then, the localization by confocal microscopy of PTB/hnRNP1 in mock-infected cells was observed mainly in the nucleus (Fig. 3Cb), and upon HAstV-8 infection PTB/hnRNP1 was distributed to the cytoplasm (Fig. 3Cf). This redistribution of PTB/hnRNP1 is due to PTB/hnRNP1 phosphorylation at Ser-16 which modulates the nucleo-cytoplasmic distribution [44], in an analogous fashion of nsp1a/4 phosphorylation that capacitates nsp1a/4-RNA polymerase interaction for the establishment a productive infection by HAstV-8 [6]. Positive HAstV-8 infected cells were monitored by the typical fluorescence signal of the VP34 capsid protein distributed throughout the cytoplasm as prominent accumulation of bright punctate granules around the nuclear membrane (Fig. 3Cg) as previously described [4]. As expected, VP34 HAstV-8 capsid protein was not detected in mock infected CaCo cells (Fig. 3Cc).

To confirm that PTB/hnRNP1 binds to HAstV8 3′UTR, UV cross-linking immunoprecipitation experiments were carried out using infected S10 CaCo2 extracts and labeled RNA. A 57/60 kDa polypeptide was precipitated with protein G-Sepharose and hnRNP1 antibody (Fig. 3D, compare to the input lane 2 with lane 8), suggesting the presence of PTB/hnRNP1 in the RNA-protein complex formed by astrovirus 3′UTR. No proteins were detected in the precipitation controls (anti-hnRNP1 antibody only, lane 3; non-related antibody, lane 4; protein G-Sepharose only, lane 6; or non-related antibody coupled to protein G-Sepharose, lane 5) and the mutant probe control, mPTB – an RNA carrying a CGAA sequence mutation in the PTB-binding linker site (Fig. 3, lane 7). Again, these results suggest that RNA-protein complex formation appears to require the presence of an intact PTB/hnRNP1binding site.

**Figure 4:**
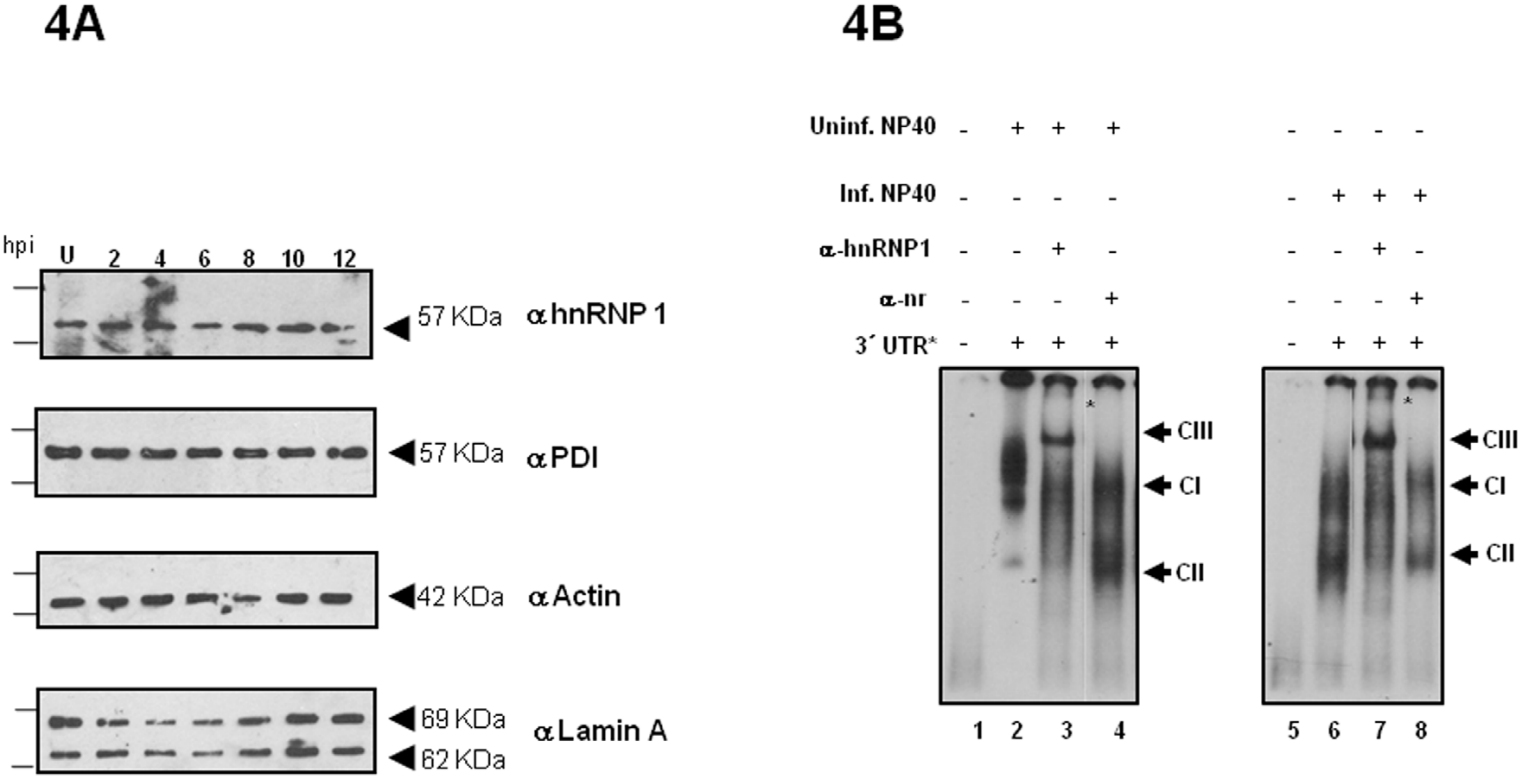
PTB is found in HAstV-8-infected CaCo2 NP-40 cell extracts. **A)** Western blot were probed with polyclonal anti-hnRNP1, anti-Actin, anti-PDI and anti-Lamin A antibodies. Sixty micrograms of uninfected (U) and infected CaCo2 cells NP-40 extracts (from 2 up to 12 hours post infection, h.p.i.) were run on SDS-PAGE and blotted onto nitrocellulose. **B)** Supermobility shift assay from uninfected or HAstV-8 infected CaCo2 NP-40 cell extracts were incubated without (lane 2) or with anti-hnRNP1 (lane 3) or with a non-related antibody (lane 4) after of the 3′UTR labeled RNA (lane 1). Supershifted complexes are indicated as CIII.

Many positive-strand RNA viruses replicate and translate in the cytoplasm, which take place in association with intracellular membranes. For example, viral replicase complexes from other positive-strand RNA viruses assemble on intracellular membrane surfaces [10], [19], around the viral RNA comprising viral replication proteins and coopted host proteins, including PTB/hnRNP1. Therefore, we explored the presence of PTB/hnRNP1 in cytoplasmic membrane-associated proteins from uninfected and HAstV-8 infected cells by western-blot assays carried out with hnRNP1 antibodies and NP40 membrane extracts obtained at different hours post infection (h p.i.). In both extracts, PTB/hnRNP1 was detected in all p.i. time points (Fig. 4A). Positive membrane fractionation and cytoplasmic and nuclear markers were assessed with anti-PDI, anti-Actin and anti-Lamin A antibodies, respectively. Additional supershifts were carried out with cytoplasmic membrane fractions as described above. The results showed a similar pattern than the S10 cytoplasmic preparation. Interestingly the intensity of all complexes signal was stronger (Fig. 4B). These results suggested that PTB/hnRNP1, cellular proteins and probably viral proteins, from membrane fractions were sufficient for 3′UTR HAstV-8 RNA-protein complex formation.

**Figure 5:**
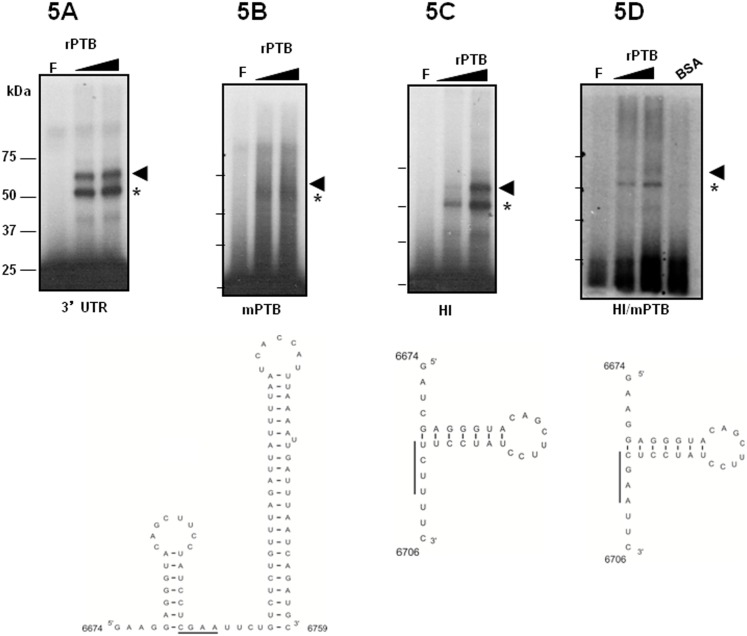
His-PTB binds to the HAstV-8 3′UTR. UV crosslinking assay carried out with radiolabeled HAstV-8 3′UTR (**A**), mPTB (**B**), HI (**C**) and HI/mPTB (**D**) were incubated with 0.50 and 1 µg of recombinant His-PTB or 1 µg of BSA (C). Free probes are shown in lanes 1 and F, respectively. The arrow and asterisk indicate the His-PTB protein cross-linked to the viral RNA. The molecular mass markers (MWM) are shown. The secondary structures of the RNA probes are shown below. Consensus PTB site is indicated as continuous line in HI, PTB sequence changes appear undelined in probes HI/mPTB and mPTB.

PTB/hnRNP1 is one of the most frequently IRES-specific cellular trans-acting factors implicated in RNA stability, translation and replication of several human viruses as poliovirus, dengue, hepatitis C (HCV) and other positive-strand RNA viruses [47], [48]. For example, in picornavirus, HCV and Feline calicivirus, PTB/hnRNP1 may act as an RNA chaperone facilitating the replication-translation switch [49], [50]. To delineate the minimal protein-interacting region of the HAstV-8 3′UTR and to explore whether Helix I or Helix II are required in this interaction, radiolabeled wt and mutant 3′UTR probes (shown in Fig. 2A) were used in UV cross-linking experiments with recombinant His-PTB. The wt 3′UTR probe rendered strong 60 and 62 kDa bands (Fig. 5A) that disappeared when the UCUU PTB binding site was mutated to CGAA (Fig. 5B). Whereas rPTB was able to bind to HI probe lacking Helix II, rendering the same two 60 and 62 kDa bands (Fig. 5C), rPTB signals were barely apparent when probe H1/mPTB was used (Fig. 5D). Since probe HI rendered almost the same results as the wt probe, we suggest that the additional CUCU PTB binding localized on Helix II does not contribute to PTB binding. Therefore a PTB dimer binds face-to-face to the wt UCUU site, as suggested by Auweter and Allain [50]. Although we cannot rule out that the CUUCCU element could be recognized by PTB in all probes (except mPTB in which the spatial configuration of Helix II might sterically hinder its recognition), we interpret the two PTB signals as a result from distinct RNA-protein cross-link products after RNase treatment. As seen before, no cross-linking was observed with different amounts of BSA however with the unrelated RNA probe a band about 35 kDa was observed (Fig. S1). These results strongly suggest that maintaining an intact PTB site (and probably an intact Helix I as well) is important for protein recognition around position 6700, whereas the second PTB/hnRNP1 binding element is not sufficient for binding.

### PTB/hnRNP1 is required for HAstV-8 replication

**Figure 6:**
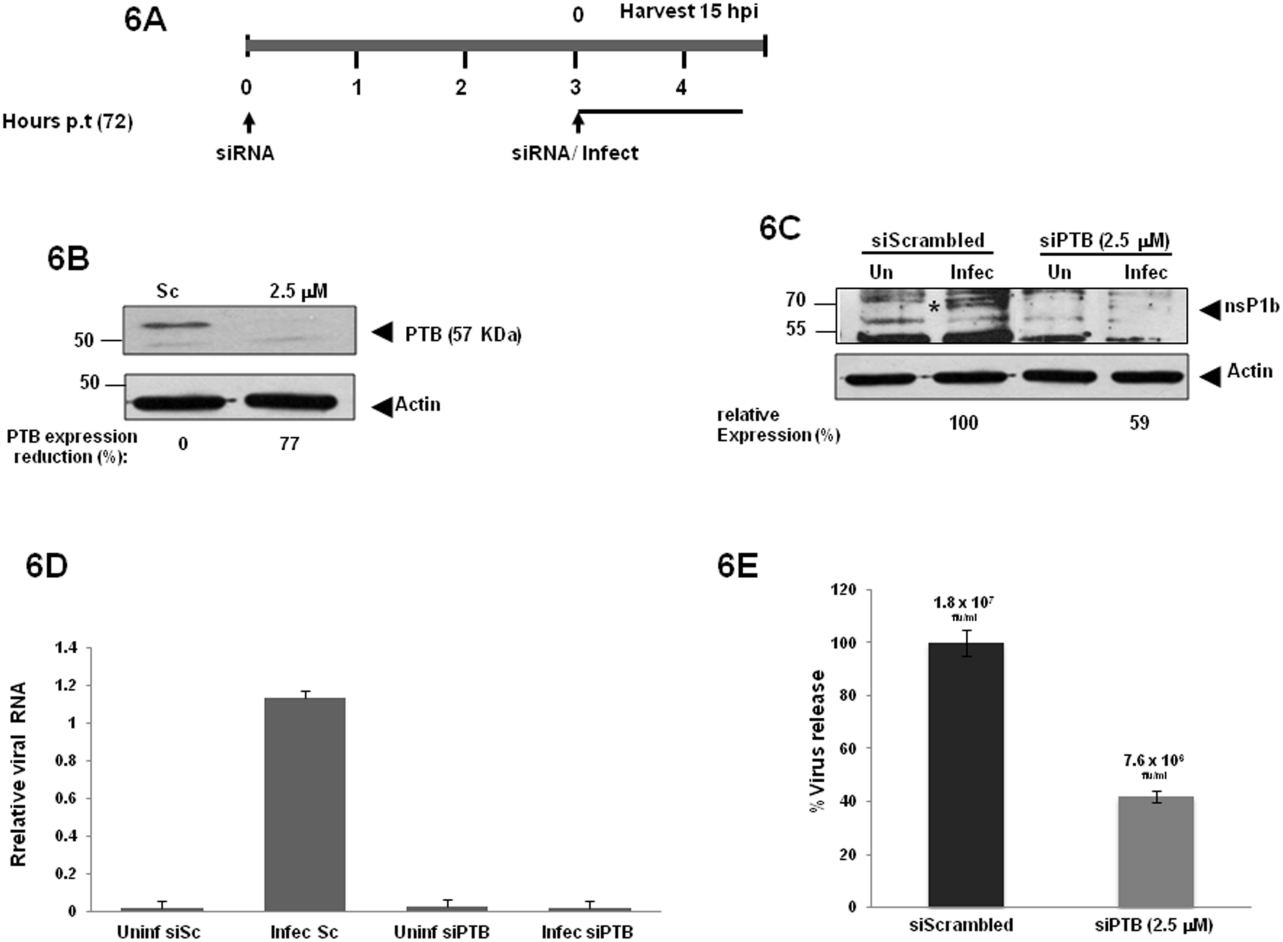
Effect of PTB knockdown on the replication of HAstV8. **A)** CaCo-2 cells were transfected with the indicated siRNA and infected with HAstV-8 following the schedule. **B)** Total protein was harvesting to demonstrate PTB knockdown by western blot, Actin was used as loading control. PTB signal intensity was quantified by densitometry. **C)** The nsP1b protein level was monitored by western-blotting in transfected and infected CaCo2 cells 15 hp.i. with polyclonal 1b-2 and actin antibodies. nsP1b signal intensity was quantified by densitometry as describe above. Signal intensity is expressed as percent of expression reduction with respect to actin signals. **D)** Relative RNA level of HAstV-8 in transfected and an infected cell were determined by qRT-PCR. The results shown are from triplicate assays; error bars represent standard deviations (*P*<0.0001, two-tailed Student *t* test). **E)** The viral yield was determined by focus forming units per ml (ffu/ml) from supernatants recovered 72 h post-transfection of CaCo-2 cells, with the indicated siRNA and HAstV-8 infection (15 hp.i.). The Y axis represents the virus release percentage of 15 hours post infection after 72 hours post-transfection time. Duplicate experiments rendered identical results.

As mentioned above, human astrovirus replication occurs within the replication complexes formed in the cytoplasm. Also, PTB/hnRNP1 is mainly located in the nucleus, although it can shuttle to the cytoplasm upon viral infections. In order to analyze a possible role of PTB/hnRNP1 in HAstV-8 replication, uninfected CaCo2 cells were first transfected with siRNAs against PTB, and the levels of PTB/hnRNP1 expression were monitored by western-blot and immunofluorescence analysis. PTB/hnRNP1 was detected as a 57/60 kDa doublet, suggesting that at least two PTB/hnRNP1 isoforms are present in CaCo2 cells (Fig. S2A), and depending on the siRNA concentration used, the expression of both PTB/hnRNP1 isoforms was reduced at least 10-fold, respectively, whereas PTB/hnRNP1 expression was unaffected in mock and scrambled-siRNA transfections (Fig. S2B). PTB/hnRNP1 knockdown was confirmed by IF also. As expected, PTB was observed in the nuclei of transfected CaCo2 cells with the scrambled siRNA. In contrast, PTB was absent from the nuclei of siPTB treated cells (Fig. S2C). PTB knockdown-cells and control-cells were then infected with HAstV-8; the samples were harvested following the protocol depicted in Fig. 6A. We first showed that siPTB-treatment for 72 h was sufficient for sustained PTB silencing without apparent cell morphological perturbance (Fig. S2). Then, compared to the scrambled siRNA (land Sc), a 77% reduction of PTB protein expression was achieved with 2.5 µM of siPTB (Fig. 6B). To understand whether PTB/hnRNP1 knockdown expression affects HAstV-8 RNA replication, the viral RNA polymerase (nsP1b) protein expression and viral RNA were determined by Western blotting and qRT-PCR, respectively (Fig. 6C and D). As expected, viral nsP1b expression was lower in 2.5 µM siPTB-treated than siScrambled-treated cells (Fig. 6C). The level of viral negative strand RNA in cells transfected with siPTB was reduced dramatically (∽90%), in comparison with siScrambled (Fig. 6D, bars Infec Sc and Infec siPTB), indicating that silencing of PTB induced a reduction in viral replication. The transfected uninfected cells were used as negative control (bars Uninf siSc and Uninf siPTB). Furthermore, reduced viral production in the culture supernatants (monitored as in references [20], [25], [26]) was observed upon PTB knockdown (Fig. 6E). The siRNA treated cells resulted in a 42% (2.35-fold/2.5 µM) reduction of viral production (7×10^6^ ffu/ml) with siPTB in comparison with siScrambled-transfected cells (1.8×10^7^ ffu/ml) (monitored as reported in reference [52]). This result is consistent with the knockdown of host cell proteases, which result in less than one log viral release reduction reported previously [51]; however our system allowed more reproducible results. A possible explanation for this modest viral particle reduction relies in the fact that the astroviral RNA negative strand synthesis appears 12 hours post-infection, coincident with the detection of the positive strand RNA and the ORF2 subgenomic RNA (sgRNA, encoding the capsid proteins). Notably at this time point, the amount of plus-sense sgRNA is tenfold greater than the minus-sense replicative intermediary RNA [52], which is translated into capsid proteins used for packaging of new viral particles [37]. It is possible that in the time frame used in our experiments, which overlap the HAstVs viral particles release (12 to 24 hpi), we were able to detect newly packaged viral particles whose RNA molecules escaped or were synthesized before PTB knockdown.

In conclusion we have shown that PTB/hnRNP1 binds to the 3′UTR of HAstV-8, recognizing the cognate binding site UCUU (localized in the linker) and helix I of the 3′UTR structure. PTB/hnRNP1 is redistributed to the cytoplasm during HAstV-8 infection in membrane-associated fractions, and it is required, directly or indirectly, for HAstV-8 replication. We are currently investigating other factors involved in such process.

## Supporting Information

Figure S1 **UV-cross linking was carried out with radiolabeled an unrelated RNA was incubated with 1 µg of recombinant His-PTB (A) or 0.25, 0.50, and 1 µg of BSA were incubated with the wild type 3′UTR RNA (B).** Free probes appear in lanes 2 and 3 respectively. The molecular mass markers (MWM) are shown.
(TIF)

Figure S2 **Caco-2 cells were mock-transfected (M) or transfected with the indicated siRNA (A).** Total protein was harvesting to demonstrate PTB knockdown by western blot, Actin was used as loading control. The reduction of PTB/hnRNP1 expression was quantitating by densitometry **(B)** taking actin or/and mock transfected cell as the reference, arbitrary density units (vertical axis) was plotted against the siRNA concentration (horizontal axis). The PTB/hnRNP1 knockdown was verified by confocal immunomicroscopy Olympus Fv300 **(C)**.
(TIF)
