# Supplementary figures and images for: PTB Binds to the 3’ Untranslated Region of the Human Astrovirus Type 8: A Possible Role in Viral Replication
Source: PLoS One. 2014 Nov 18;9(11):e113113. doi: 10.1371/journal.pone.0113113 (PMC4236132; doi:10.1371/journal.pone.0113113)

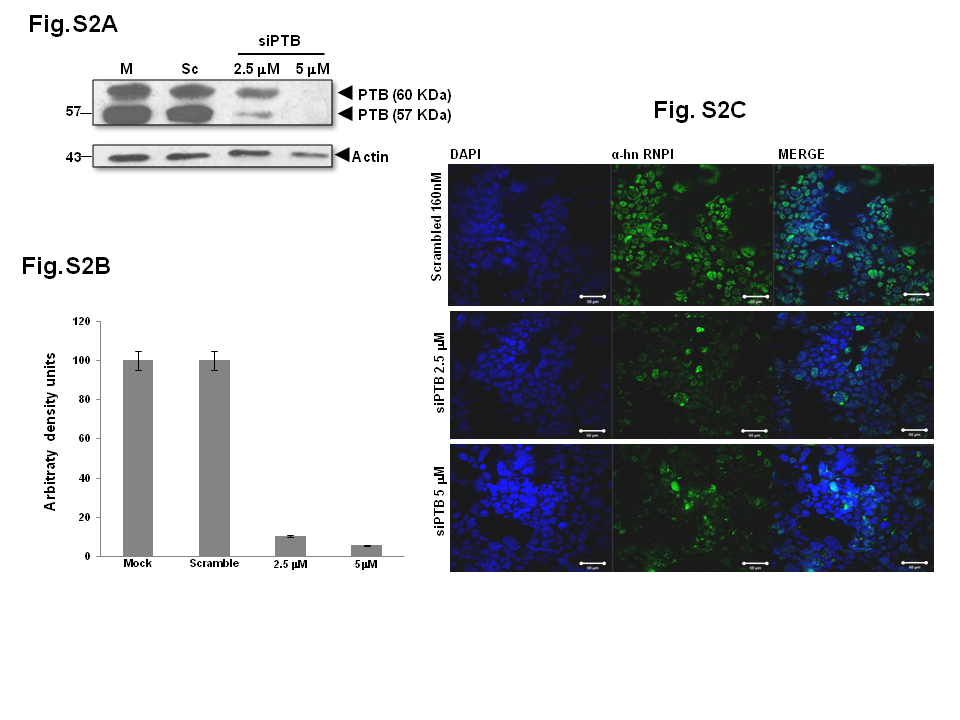

Supplement: Figure S2 — Caco-2 cells were mock-transfected (M) or transfected with the indicated siRNA (A). Total protein was harvesting to demonstrate PTB knockdown by western blot, Actin was used as loading control. The reduction of PTB/hnRNP1 expression was quantitating by densitometry (B) taking actin or/and mock transfected cell as the reference, arbitrary density units (vertical axis) was plotted against the siRNA concentration (horizontal axis). The PTB/hnRNP1 knockdown was verified by confocal immunomicroscopy Olympus Fv300 (C). (TIF) [file pone.0113113.s002.tif]
